# Severe Hypercalcemia and Acute Renal Failure: An Unusual Presentation of Sarcoidosis

**DOI:** 10.1155/2010/423659

**Published:** 2010-12-16

**Authors:** Rudruidee Karnchanasorn, Molly Sarikonda, Saleh Aldasouqi, Ved V. Gossain

**Affiliations:** ^1^Department of Diabetes, Endocrinology & Metabolism, City of Hope, University of California, 1500 East Duarte Road, Duarte, CA 91010, USA; ^2^Department of Internal Medicine - Infectious Diseases, Washington University, Campus Box 8051, St. Louis, MO 63130, USA; ^3^Michigan State University, East Lansing, MI 48824, USA

## Abstract

Although hypercalcemia is a known metabolic complication of sarcoidosis, it is rarely a presenting manifestation. Long-standing hypercalcemia and hypercalciuria can cause nephrocalcinosis and chronic renal failure. Acute renal failure, although described, is also a rare presentation of patients with sarcoidosis. We describe two patients with sarcoidosis, who presented with severe hypercalcemia and worsening renal function. Parathyroid hormone levels were appropriately suppressed. This led to an extensive search for the cause of hypercalcemia. Finally, after a lymph node biopsy in both cases, a diagnosis of sarcoidosis was established, hypercalcemia resolved, and renal function improved in both cases after administration of prednisone.

## 1. Introduction

Although hypercalcemia is a known metabolic complication of sarcoidosis, it is rarely a presenting manifestation. Hypercalciuria is the most common defect of calcium metabolism with a prevalence of 50–62% [1–3]. Although hypercalcemia in 11% and renal calculi in 10% of patients with sarcoidosis has been reported [4], clinically significant hypercalcemia is less frequent and is generally asymptomatic, occurring in less than 5% of patients [5]. Persistent untreated hypercalcemia and hypercalciuria can lead to nephrocalcinosis, renal calculi, and renal failure [6]. We report two cases of sarcoidosis, where the initial presentation was markedly elevated serum calcium levels and acute renal failure, which presented a diagnostic challenge.

## 2. Case  1

**Figure 1:**
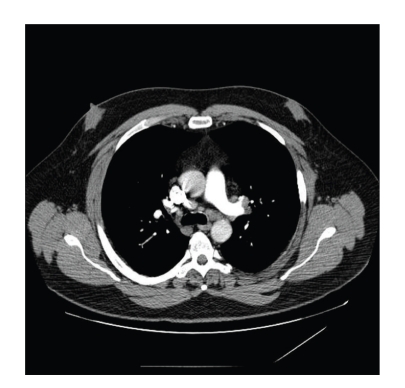
CT Chest showing mediastinal lymphadenopathy.

**Figure 2:**
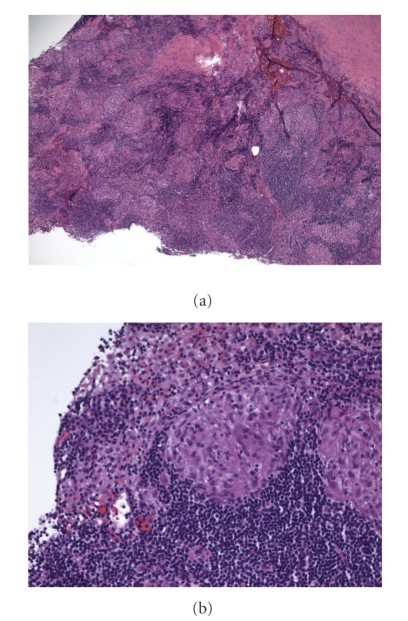
(a) Lymph node biopsy (low power). (b) Lymph node biopsy (high power).

 A 56-year-old Caucasian male was admitted three times to the hospital in 3 months for recurrent abdominal pain. He described it as an intermittent cramping pain, located in the epigastrium, with some radiation to the left lower quadrant. He also experienced intermittent nausea, vomiting, and watery diarrhea. The pain was aggravated by lying in supine position and improved with sitting upright. He denied having any fever, chills, or any other associated symptoms. He also noted excessive thirst and increased in urination but denied dysuria or hematuria. He was found to have persistent hypercalcemia and renal insufficiency (see Table 1). A review of his medical record revealed that he had been admitted to the hospital twice during the preceding two months with similar symptomatology. Each time he was noted to have hypercalcemia (serum calcium 13.3 and 13.5 mg/dL, resp.) and acute renal insufficiency (BUN 44 mg/dL and creatinine 2.1 mg/dL). Serum and urine protein electrophoresis were negative. 1,25-dihydroxyvitamin D level was 69 ng/mL (reference range 22–67 ng/mL), and a simultaneously obtained PTH level was 6.9 pg/mL (reference range 14–72 pg/mL). His past medical history was only significant for nephrolithiasis. He was not taking any medications regularly. Physical examination revealed blood pressure of 129/80 mmHg, heart rate 80/min regular. He was febrile and did not appear to be in acute distress. His cardiopulmonary exam was unremarkable. Abdominal exam revealed mild tenderness in the epigastrium area and left lower quadrant without any signs of guarding or rigidity. Bowel sounds were active. Acute abdominal series X-ray did not show any acute process. Laboratory investigation showed normal CBC and urine analysis. Peak serum calcium was 13.5 mg/dL (reference range 8–10 mg/dL), and ionized calcium was elevated at 1.68 mmol/L (reference range 1.10–1.30 mmol/L). Serum creatinine level was 3.4 mg/dL (0.5–1.4 mg/dL). 1,25-dihydroxyvitamin D levels were elevated and parathyroid hormone (PTH) levels were appropriately suppressed (see Table 1). Serum angiotensin converting enzyme level was elevated at 98 units/L (reference range 8–52 units/L). He was treated with intravenous fluid hydration and loop diuretic while the etiology of hypercalcemia was being sought, including an extensive investigation for occult malignancy and granulomatous diseases. Chest X-ray was normal. CT scans of chest, abdomen, and pelvis were performed. Bone scan was unremarkable. CT Chest revealed subtle mediastinal adenopathy (Figure 1), and subsequent biopsy (Figures 2(a) and 2(b)) confirmed the diagnosis of sarcoidosis. Corticosteroid therapy was initiated and hypercalcemia eventually resolved, and renal function also improved (BUN 32, creatinine 1.8 mg/dL).

## 3. Case  2

**Figure 3:**
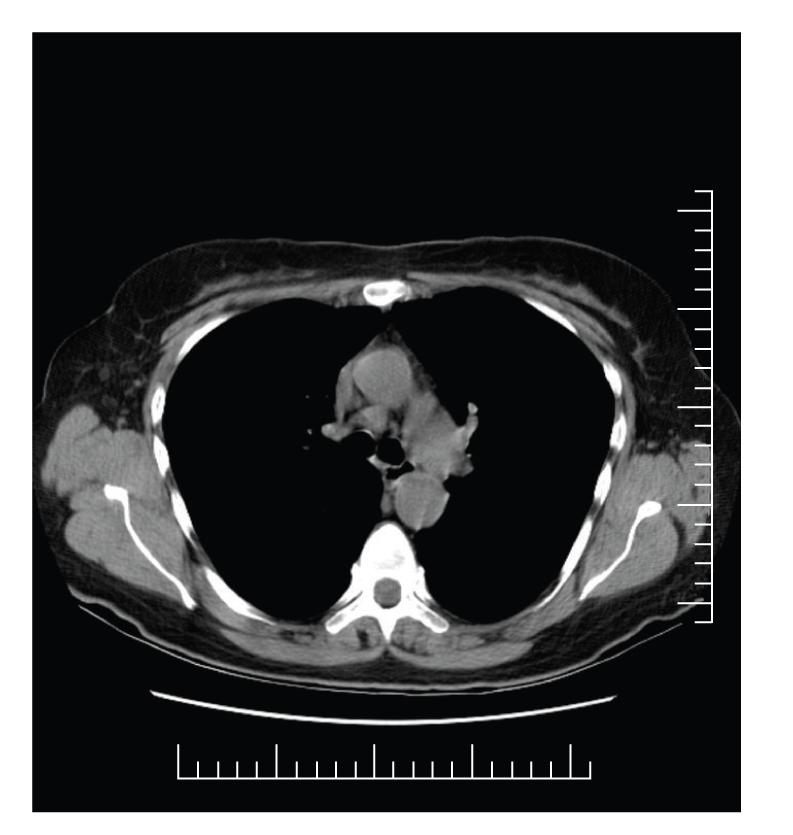
CT Chest showing mediastinal lymphadenopathy.

**Figure 4:**
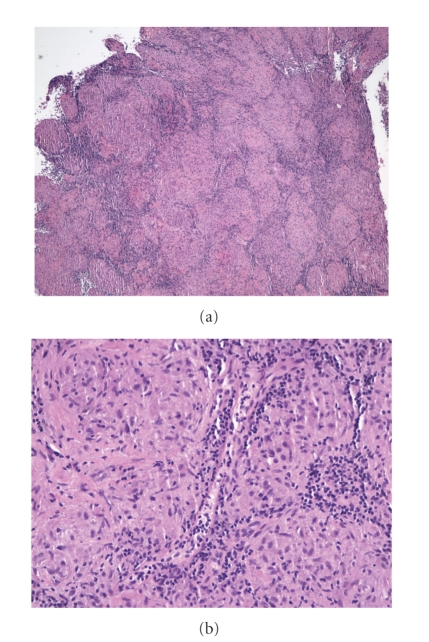
(a) Lymph node biopsy showing noncaseating granulomatous change (low power). (b) Lymph node biopsy showing noncaseating granulomatous change (high power).

 A 59-year-old Caucasian female was incidentally noted to have hypercalcemia and elevated blood pressure during routine outpatient labs screening. She stated that she had been feeling well. She denied any history of fatigue, nausea, anorexia, constipation, muscle ache, or changes in sensorium. She did not complain of polyuria or polydipsia. However, she stated that she drank 32 to 44 ounces of water during the day as a matter of habit. She had lost 25 pounds of weight within the past six months unintentionally. She had a remote history of nephrolithiasis in 1960 and occasional bilateral hip pain. She also gave a history of removal of a skin lesion on the nose, which was reported as “benign.” She denied taking any vitamin or other herbal supplements. There was no family history of endocrine disorders. She had no history of smoking tobacco, consuming alcohol, or use of illicit drugs. She had not been taking any medications. Physical examination revealed elevated blood pressure of 210/98 mmHg that substantially came down to 146/86 with treatment. Oral temperature was 99.4 F (37.4°C); pulse had a regular rhythm with a rate of 80 bpm. She was alert, oriented, and did not appear to be in acute distress. Her lungs were clear to auscultation with normal respiratory effort. A grade 2/6 systolic ejection murmur was heard over left sternal border. Abdomen was soft and not tender to palpation. Muscle strengths were normal and symmetrical in all extremities. She was found to have normochromic normocytic anemia with hemoglobin of 9.9 g/dL. Serum calcium level was noted to be persistently elevated over a period of 6 months with a peak level of 14.0 mg/dL (range from 11.4 to 14.0 mg/dL). Renal function was impaired with a peak serum creatinine of 2.4 mg/dL (range 1.7–2.4 mg/dL). BUN ranged from 26 to 32 mg/dL. 24 hours urine calcium was elevated at 448 mg and 587 mg measured on two different occasions (reference range 100–300 mg/ 24 hr). Serum phosphate and 25 hydroxyvitamin D levels were within normal limits (see Table 1). PTH level was appropriately suppressed and PTH related peptide (PTHrP) was elevated. A diagnosis of occult malignancy was suspected. Angiotensin-converting enzyme (ACE) was elevated (see Table 1). Chest and spine X-rays were negative as well as mammogram, bone marrow biopsy, and bone scan. Extensive evaluation failed to reveal malignancy. CT scan of the chest was performed as part of a malignancy workup, and it showed borderline mediastinal adenopathy in the paraaortic and pretracheal area and subcarinal region (Figure 3). Biopsy of the lymph nodes confirmed the diagnosis of sarcoidosis (Figures 4(a) and 4(b)). With steroid treatment, the hypercalcemia resolved (Ca = 9.2 mg/dL) and renal function also improved (BUN 21.3 mg/dL, creatinine 0.8 mg/dL). 

## 4. Discussion

Hypercalcemia is a common clinical entity encountered these days because of the routine multichannel chemistry screening. The most common cause of hypercalcemia in the outpatient clinic is primary hyperparathyroidism, whereas malignancy is the most common cause of hypercalcemia in hospitalized patients. Other causes of hypercalcemia, including sarcoidosis, are relatively uncommon. Both of our patients presented with markedly elevated serum calcium levels (13.5 and 13.3 mg/dL), and there were no other clinical clues to suggest sarcoidosis as the diagnosis. Both patients had appropriately suppressed PTH levels suggesting that the cause of hypercalcemia was not parathyroid hormone dependent. Patient 2 also had an elevated PTHrP which suggested an increased likelihood for malignancy. Both patients underwent an extensive, but futile search for malignancy. 

Sarcoidosis is an idiopathic, multisystem, and granulomatous disease. It affects people of all racial and ethnic groups of all ages. Its peak incidence is between 20 and 39 years. The incidence among African Americans is approximately three times than among white Americans and tends to occur later in life [4].

The fundamental abnormality in sarcoidosis involves the formation of immune granulomas in various organs. The lung and the intrathoracic lymph nodes are the main organ systems involved, but every organ may be affected [7]. The frequent extrathoracic involvement sites include peripheral lymph nodes, eyes, skin, and liver, each being found in approximately 10–25% of cases [7]. Sarcoidosis commonly presents with the abnormalities detected on a chest X-ray (8–60%), but the presentation may vary depending on several factors such as age, sex, race, the duration of the disease, and the sites of involvement. European population is more likely to present asymptomatically or with erythema nodosum, while symptomatic and multivisceral presentations are common in African Americans. The initial symptoms of presentation include respiratory symptoms in 30% cases, constitutional symptoms such as fatigue (27%), weight loss (28%), fever (10–27%), and erythema nodosum (3–44%) [7]. Lofgren's syndrome is an acute form of disease, consisting of arthritis, erythema nodosum, and bilateral hilar adenopathy (9–34%) and presents differently in men and women [4].

As mentioned above, although hypercalcemia occurs in about 11% cases of sarcoidosis [4], clinically significant hypercalcemia is less common [6], and we believe even rarer as the presenting feature. However, we were unable to determine how often it is the presenting symptom. Similarly, although acute renal failure as the presenting feature of sarcoidosis has been described, it remains of rare occurrence [8, 9].

The mechanism of hypercalcemia in sarcoidosis is not completely understood. Normally, the levels of vitamin D and its active metabolite 1,25-dihydroxyvitamin D play an important role in the maintenance of serum CA^++^ levels. 

Vitamin D is derived either from endogenous production of vitamin D in the skin or by ingestion of vitamin D. Vitamin D is hydroxylated to 25 hydroxyvitamin D in the liver, which is subsequently converted to 1,25-dihydroxy D in the proximal renal tubules by the enzyme 1, *α* hydroxylase. This enzyme is tightly regulated by parathyroid hormone (PTH), serum calcium, and phosphorus levels [10].

In 1939, Harrell and Fisher reported the occurrence of hypercalcemia in 6 of 11 patients with sarcoidosis [11]. On subsequent studies, in 1979, Papapoulos et al. [12] and Bell et al. [13] were among the first to recognize that levels of 1,25-dihydroxyvitamin D are elevated in patients with sarcoidosis. The high levels of 1,25-dihydroxyvitamin D are the probable cause of hypercalcemia, but overproduction of bone resorbing cytokines and PTHrP may also play a role [14]. The level of PTHrP in one of our patients was elevated, but in case 1 it was normal. Zeimer et al. described two cases of sarcoidosis with hypercalcemia and elevated PTHrP. They also demonstrated that in an archival study of 20 lymph node biopsies with the pathological diagnosis of sarcoidosis, where immunohistochemical analysis detected PTHrP in macrophages with granulomata in 17 out of 20 biopsies (85%) [14]. The renal 1, *α* hydroxylase is activated by PTH and suppressed by 1,25-dihydroxyvitamin D [10]. The macrophage enzyme is unaffected by any of these factors. Instead, one of its major trophic activators is cytokine interferon gamma [15]. Lack of feedback in response to increased levels of 1,25-dihydroxyvitamin D leads to an enhanced calcium absorption in the small intestine and an excess of bone resorption in patients with sarcoidosis, leading to hypercalcemia.

 The diagnosis of sarcoidosis may be elusive, especially in atypical populations, given the variation of the presentation of the organs involved. Hypercalcemia may be a clue to the diagnosis, especially in cases with no pulmonary symptoms or findings on chest X-ray. Sarcoidosis should be considered in patients with hypercalcemia, low PTH levels, and elevated calcitriol levels. As mentioned above, longstanding hypercalcemia and hypercalciuria can cause nephrocalcinosis and renal failure [2, 6]. Although severe hypercalcemia is rare in sarcoidosis [1, 5], patients may present with severe hypercalcemia, associated with renal failure, as was the case in our patients. 

Treatment of hypercalcemia due to sarcoidosis is beyond the scope of this paper. However, it has been reviewed by Conron et al. [1] and Sharma [16] and is discussed here only briefly. Therapeutic options include rehydration, a loop diuretic to promote calcium secretion, and corticosteroids [1]. Corticosteroids have been the first-line therapy in treating hypercalcemia associated with sarcoidosis because of their effectiveness in rapidly correcting hypercalcemia.

Prednisone in 20 to 40 mg/day is the drug of choice to reduce the endogenous production of 1,25 dihydroxyvitamin D. Institution of steroids causes a relatively swift decrease in circulating 1,25 dihydroxyvitamin D and serum calcium levels in 3 to 5 days [16]. Patients with asymptomatic mild hypercalcemia should be advised to avoid sun exposure and vitamin D rich diet and maintain hydration of more than 2 liters per day. The role of avoiding dairy product and low calcium diet is controversial since there is little evidence that it affects calcium balance [1].

Ketoconazole is an alternative for patients where the steroid use is contraindicated. It inhibits the cytochrome P450-linked enzyme systems involved in steroid synthesis including 25(OH) D_3_-1a-hydroxylase [17]. Hydroxychloroquine also causes similar effects and can be used in patients who cannot tolerate ketoconazole or who develop abnormal liver function tests [18]. Methotrexate and azathioprine may also help control hypercalcemia by reducing the granuloma formation [1].

## 5. Conclusion

We report two patients with sarcoidosis, where severe hypercalcemia associated with acute renal failure was the unusual presenting feature. It was only after extensive investigation that a diagnosis of sarcoidosis was confirmed. Sarcoidosis without pulmonary symptoms may pose a diagnostic challenge but should be suspected in patients with nonparathyroid-dependent hypercalcemia. Timely recognition and appropriate treatment of hypercalcemia secondary to sarcoidosis should prevent development of nephrocalcinosis and renal failure.

## Figures and Tables

**Table 1:** Laboratory values of patients 1 and 2.

	Normal value	Patient no. 1	Patient no. 2
Calcium	8–10.5 mg/dL	13.5 mg/dL	13.3 mg/dL
Ionized calcium	1.10–1.30 mmol/L	1.68 mmol/L	1.66 mmol/L
Serum creatinine	0.5–1.4 mg/dL	3.4 mg/dL	2.4 mg/dL
BUN	6–23 mg/dL	55 mg/dL	32 mg/dL
25 hydroxyvitamin D	14–42 ng/mL	22 ng/mL	21 ng/mL
1,25- Dihydroxyvitamin D	22–67 ng/mL	69 ng/L	Not done
Alkaline phosphatase	1–120 U/L	44 U/L	48 U/L
Phosphorus	2.5–4.5 mg/dL	3.4 mg/dL	3.1 mg/dL
Parathyroid hormone	12–72 pg/mL	9.1 pg/mL	9.0 pg/mL
PTHrP	<0.2 pmol/L	<0.2 pmol/L	0.4 pmol/L
ACE level	8–52 U/L	98 U/L	71 U/L
24° urinary calcium	100–300 mg/24°	1074	448 and 587*
TSH	0.35–5.50 uIU/mL	1.80 uIU/mL	2.8 mIU/mL
CBC	HGB 12–15 g/dL	HGB 12.8 g/dL	HGB 9.9 g/dL
	HCT 36–45%	HCT 34.6%	HCT 29.4%
	WBC 4–12 × 10^3^/mm^3^g/dL	WBC 5.6 × 10^3^/mm^3^	WBC 4.1 × 10^3^/mm^3^
	Platelets 150–400 g/dL	Platelets 132 g/dL	Platelets 190 g/dL

*Measured on two different occasions.
